# Finding a needle in a haystack: The identification of clinical practice guidelines for psychosocial oncology through an environmental scan of the academic and gray literature

**DOI:** 10.1002/cam4.7039

**Published:** 2024-02-24

**Authors:** Catherine Bergeron, Michelle Azzi, Adina Coroiu, Carmen G. Loiselle, Martin Drapeau, Annett Körner

**Affiliations:** ^1^ Department of Educational and Counselling Psychology McGill University Montreal Quebec Canada; ^2^ Centre for Addiction and Mental Health Toronto Ontario Canada; ^3^ Ingram School of Nursing McGill University Montreal Canada; ^4^ Department of Oncology McGill University Montreal Quebec Canada; ^5^ Lady Davis Institute for Medical Research, Jewish General Hospital Montreal Quebec Canada; ^6^ Louise Granofsky Psychosocial Oncology Program, Segal Cancer Centre Montreal Quebec Canada; ^7^ Psychosocial Oncology Program McGill University Health Centre Montreal Quebec Canada

**Keywords:** clinical practice guidelines, evidence‐based practice, psychosocial oncology, supportive care for cancer

## Abstract

**Objective:**

Clinical practice guidelines (CPGs) are evidence‐based tools well‐suited to translate the latest research evidence into recommendations for routine clinical care. Given the rapid expansion of psychosocial oncology research, they represent a key opportunity for informing the treatment decisions of overburdened clinicians, standardizing service delivery, and improving patient‐reported outcomes. Yet, there is little consensus on how clinicians can most effectively access these tools and little to no information on the current availability and scope of CPGs for the range of psychosocial symptoms and concerns experienced by patients with cancer.

**Method:**

Our environmental scan consisted of an academic and gray literature designed to identify currently available CPGs addressing a range of cancer‐related psychosocial symptoms.

**Results:**

Findings revealed a total of 23 existing psychosocial oncology CPGs that met full eligibility criteria. The gray literature search was found to be more effective at identifying CPGs (*n* = 22) compared to the academic search (*n* = 9).

**Conclusion:**

Several concerns arose from the systematic search. The limited publication of CPGs in peer‐reviewed journals may make clinicians and stakeholders more hesitant to implement CPGs due to uncertainties about the methodological rigor of the development process. Further, many existing CPGs are outdated or failed to be updated according to guideline recommendations, meaning that the recommendations may fall short of their purpose to translate up‐to‐date research findings.

**Future directions:**

Future research should seek to systematically assess the quality of existing psychosocial oncology CPGs and shed light on the current state of implementation and adherence in clinical practice in order to better inform guideline developers on the current needs of the psychosocial oncology community.

## 1 INTRODUCTION

Cancer is one of the leading causes of death across the world, with as many as 19.3 million new cases diagnosed worldwide in 2020 alone.^1^ As incidence rates are expected to rise by nearly 60% over the next 20 years,^2^ the burden of this disease will also increase, with an unprecedented number of individuals, families, and communities facing not only the associated physical ailments and escalating costs incurred by healthcare systems, but also a wide range of psychological, social, and occupational challenges.^2^, ^3^ Common psychosocial symptoms reported by patients with cancer include overall psychological distress,^4^, ^5^, ^6^ symptoms of depression^6^, ^7^ and anxiety,^6^, ^8^ demoralization,^9^ interpersonal difficulties,^5^, ^10^ and reduced quality of life.^11^

In 2007, the US Institute of Medicine recommended that treating psychosocial symptoms in patients with cancer is best practice for comprehensive cancer care.^12^, ^13^ This assertion has been widely endorsed, as evidenced by the rapid advances in the availability of new psychosocial oncology services, upsurges in relevant peer‐reviewed articles, care standards, and reports, and the development of new interventions.^2^, ^14^ The rapid expansion of psychosocial oncology poses a challenge for clinicians providing day‐to‐day clinical care. Psychosocial oncology clinicians experience large caseloads, have limited time for selecting and reviewing relevant scientific literature, and might have limited capacity to translate empirical knowledge into practical interventions to be delivered at the point of care.^15^, ^16^, ^17^

Clinical practice guidelines (CPGs) are well‐suited to bridge the gap between scholarly knowledge and service delivery. CPGs are systematically developed tools designed to summarize the latest scientific evidence and provide clinically applicable recommendations to inform treatment decisions for various health conditions.^18^ CPGs targeting psychosocial symptoms for patients with cancer provide recommendations based on the latest scientific findings for clinical care at all stages of the cancer care continuum, from screening to treatment and follow‐up, and also help standardize service offerings and optimize the use of often limited resources.^19^, ^20^, ^21^ Yet, little is known about the current scope and accessibility of CPGs in psychosocial oncology. Currently, there is no universally accessible, comprehensive repository for CPGs, which makes it difficult for clinician to find and access such evidence‐based documents. While some CPGs may be accessible through “traditional” academic sources (e.g., PubMed database), clinicians may lack institutional access to databases depending on their work settings. Further, some CPGs are not indexed in academic databases and can only be accessed through non‐commercial sources.^22^ For example, certain special interest groups and government‐funded organizations only publish their CPGs on their organizational websites.

### 1.1 Study objective

To bridge this knowledge gap, we conducted an environmental scan to identify the number and scope of clinical practice guidelines that target the psychosocial symptoms of individuals diagnosed with cancer across the cancer care trajectory and examine their accessibility through different search methods.

## 2 METHODS

### 2.1 Procedures

An environmental scan is a type of review that encompasses both published literature (e.g., peer‐reviewed articles) and unpublished or informally published documents.^23^ We selected this review method to increase the likelihood of identifying existing psychosocial oncology CPGs, which are often uploaded onto institutional or organizational websites by their developers. Our environmental scan was informed by the recommendations for systematic reviews for CPGs outlined by Johnston et al.^22^ We included two separate systematic searches to review (a) the academic literature and (b) the “gray” literature. Initial searches were conducted from January 2023 to February 2023 and updated in July 2023.

### 2.2 Inclusion and exclusion criteria

Eligibility criteria were established in two steps. First, we identified relevant CPGs based on methodological characteristics, as per the National Guidelines Clearinghouse 2013 revised criteria^24^ and the Institute of Medicine's 2011 definition of clinical practice guidelines,^18^ which included: (1) Systematically developed treatment decision‐making recommendations for healthcare professionals; (2) Recommendations developed or endorsed by a professional, medical, public, or private agency/organization; and (3) Full CPG text developed and/or available in English. Second, we used the **P**opulation, **I**nterventions, **C**ontent, **A**ttributes of eligible CPGs, and **R**ecommendation characteristics (PICAR) framework, which adapted the PICOTS framework to CPGs^25^: (1) Population included patients diagnosed with cancer at any disease stage; (2) Recommendations targeted at least one psychosocial aspect of cancer; (3) Recommendations for the treatment of psychosocial symptoms focused on non‐pharmacological interventions; and (4) Recommendations were published, revised, and/or updated within the past 10 years. Supplemental guidelines developed by the same organization complementary to pre‐existing CPGs were also included.

Exclusion criteria included: (1) Reports exclusively documenting updated methodology and outcomes; (2) Consensus statements synthesizing expert and stakeholder opinions about care standards for use in contexts with minimal evidence^26^; (3) Recommendations not relevant to psychosocial oncology; (4) Over 10 years since date of publication or update; and (5) Full text and/or recommendations not available in English.

### 2.3 Search strategy

#### 2.3.1 Academic literature search

This search consisted of a systematic search of five peer‐reviewed academic databases (PubMed, Medline, JSTOR, PsycINFO, and Clinical Key) and was developed in consultation with a research librarian at McGill University. Relevant keywords for each applicable PICAR component were identified through a review of the literature on psychosocial oncology care and in consultation with experts and our research team. Keywords were utilized as either free‐text or MeSH terms (if available) and paired with Boolean operators. To maximize the retrieval of relevant results and ensure a comprehensive search, spelling variations were included through database‐specific truncation and search settings were set to identify keywords in all text (i.e., titles, abstracts, subject heading, full text, etc.). Results were filtered by language to include only English results and by publication type (i.e., guideline) when applicable. See Appendix, Table A1 for the electronic search strategy for each database.

#### 2.3.2 Gray literature search

The gray literature search included a multistep approach. Step 1 consisted of a generic Internet search using the Google search engine through the Google Chrome browser. Based on recommendations for limiting method‐inherent selection bias, cookies and search history were cleared from the browser prior to conducting the search.^27^, ^28^ Keywords and search terms from the academic search were adapted for the generic Internet search. Step 2 consisted of a Google Scholar search, which indexes both peer‐reviewed and gray literature resources.^28^ Keywords and search terms from the academic search were used to generate relevant search strings and searched within both “full text” and “title” using the advanced search functions. Step 3 consisted of a targeted Internet search for CPGs through the official websites of organizations, groups, agencies, and government bodies involved in psychosocial oncology or guideline development. The identification of relevant websites was informed by our literature search and consultation with our research team as well as domain experts. We consulted the websites of the following organizations: the National Comprehensive Cancer Network (NCCN; https://www.nccn.org/), the Canadian Association of Psychosocial Oncology (CAPO; https://www.capo.ca/), Cancer Care Ontario (CCO; https://www.cancercareontario.ca/en), the European Society for Medical Oncology (ESMO; https://www.esmo.org/), the American Society of Clinical Oncology (ASCO; https://www.asco.org/), the American Psychosocial Oncology Society (APOS; https://apos‐society.org/), and the International Psycho‐Oncology Society (IPOS; https://www.ipos‐society.org/).

### 2.4 Identification and data extraction

All results generated from the academic and targeted Internet search were retained. As both the generic Internet search and Google Scholar search were expected to produce more results than was feasible to screen, we followed the recommendation by Haddaway et al.^28^ to limit screening to the first 300 results for each search string (i.e., 30 pages). Results were imported into Zotero 6.0.22, deduplicated, and screened for eligibility. An initial screening of titles and abstracts was conducted, followed by a full‐text screening. If multiple versions of a CPG were identified, only the latest version of the CPG was retained for review. Following identification, data extracted from guidelines included: developer, location, psychosocial symptoms/concerns addressed, target population (age and cancer type), and stage of care. CPG identification and data extraction was independently conducted by two reviewers. All disagreements between the reviewers were recorded and discussed until consensus was reached.

## 3 RESULTS

### 3.1 Screening and reliability

Two reviewers independently screened the results from all search methods based on the eligibility criteria described above and identified a total of 35 psychosocial oncology CPGs. Following a consensus meeting, 14 disagreements in identification resulted in 12 excluded records and two determined to meet full eligibility criteria. Reasons for exclusion were insufficient relevance to psychosocial oncology (*n* = 7), no evidence for the use of systematic methods to develop treatment recommendations (*n* = 4), and availability of a more up‐to‐date version (*n* = 1). Reliability at the full‐text review stage was excellent^29^ between the two reviewers (Cohen's *κ* = 0.88).

### 3.2 Clinical practice guideline identification

**FIGURE 1:**
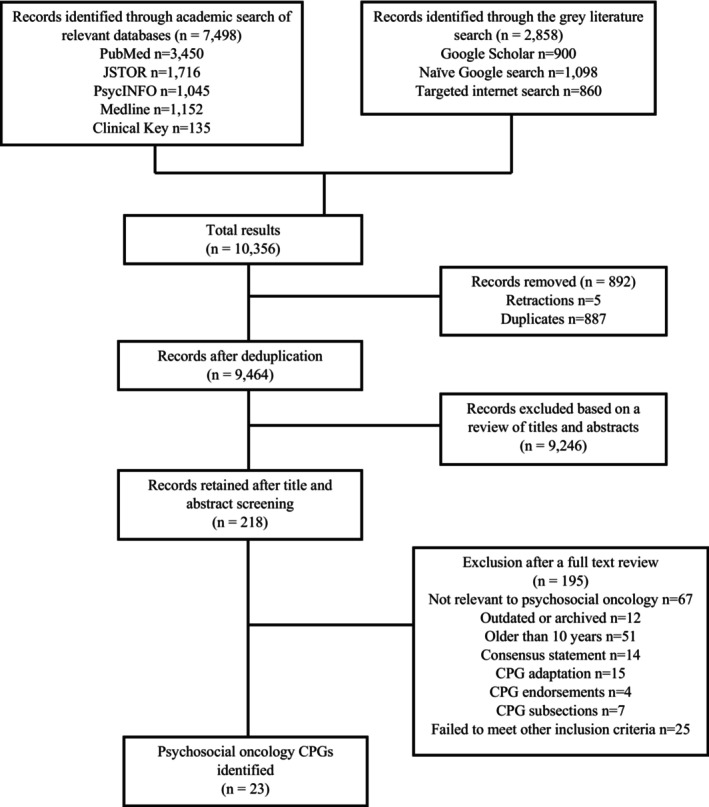
PRISMA flow diagram for the identification of CPGs.

A total of 10,423 initial records were generated by the various search methods employed in this study. Prior to screening and deduplication, five records were flagged by Retraction Watch and removed. The listed reasons for retraction included: copyright claims (*n* = 1), self‐plagiarism (*n* = 1), errors in the data (*n* = 1), problems with the results (*n* = 2), objections by a third party (*n* = 1), and withdrawal (*n* = 1). A total of 218 records were retained for a full‐text review. Based on both the methodological and PICAR eligibility criteria, 195 records were excluded following a full‐text review and reviewer consensus meetings. In total, 23 records were deemed eligible and identified as psychosocial oncology CPGs. Refer to Table 1 for a complete list of all identified CPGs. Additionally, a PRISMA flow diagram detailing the CPG selection process can be found in Figure 1.

**TABLE 1:** Characteristics of all identified psychosocial oncology clinical practice guidelines (CPGs).

Title	Year	Developer, country	Psychosocial symptoms/concerns covered	Age	Type of care	Cancer type
1. American Cancer Society Colorectal Cancer Survivorship Care Guidelines	2015	American Cancer Society, USA	Distress, depression, anxiety, fatigue, sexual dysfunction, body image, existential/spiritual concerns, social support	Adults	Follow‐up care	Colorectal cancer
2. American Cancer Society Head and Neck Cancer Survivorship Care Guideline	2016	American Cancer Society, USA	Fatigue, sleep disturbances, body image, distress, depression	Adults	Follow‐up care	Head and neck cancer
3. American Cancer Society Prostate Cancer Survivorship Care Guidelines	2014	American Cancer Society, USA	Depression, distress, anxiety, existential/spiritual concerns, body image, sexual dysfunction, social support	Adults	Follow‐up care	Prostate cancer
4. Clinical Practice Guidelines on the Evidence‐Based Use of Integrative Therapies During and After Breast Cancer Treatment	2017	American Cancer Society, USA	Anxiety, depression, fatigue, quality of life, sleep disturbances	Adults	Any stage of care	Breast cancer
5. American Cancer Society/American Society of Clinical Oncology Breast Cancer Survivorship Care Guideline	2016	American Cancer Society & American Society of Clinical Oncology (ASCO), USA	Distress, depression, anxiety, fatigue, sexual dysfunction	Adults	Follow‐up care	Breast cancer
6. Integration of Palliative Care into Standard Oncology Care: American Society of Clinical Oncology Clinical Practice Guideline Update	2017	American Society of Clinical Oncology (ASCO), USA	Social support, distress, fatigue, sexual dysfunction	Adults	Palliative care	Any cancer type
7. Management of Anxiety and Depression in Adult Survivors of Cancer	2023	American Society of Clinical Oncology (ASCO), USA	Depression, anxiety	Adults	Follow‐up care	Any cancer type
8. Practical Assessment and Management of Vulnerabilities in Older Patients Receiving Chemotherapy	2023	American Society of Clinical Oncology (ASCO), USA	Depression, social support	Older adults	Active treatment	Any cancer type
9. Palliative and End‐of‐Life Care in Lung Cancer	2013	American College of Chest Physicians, USA	Quality of life, existential/spiritual concerns, distress, social support	Adults	Palliative care	Any cancer type
10. A Pan‐Canadian Practice Guideline for Screening, Assessment, and Management of Cancer‐Related Fatigue in Adults	2015	Canadian Association of Psychosocial Oncology (CAPO), Canada	Fatigue	Adults	Any stage of care	Any cancer type
11. Pan‐Canadian Practice Guideline: Screening, Assessment and Management of Psychosocial Distress, Depression and Anxiety in Adults with Cancer	2015	Canadian Association of Psychosocial Oncology (CAPO), Canada	Distress, depression, anxiety	Adults	Any stage of care	Any cancer type
12. Management of Depression in Patients with Cancer: A Clinical Practice Guideline	2016	Cancer Care Ontario, Canada	Depression, distress	Adults	Any stage of care	Any cancer type
13. Follow‐up Care and Psychosocial Needs of Survivors of Prostate Cancer	2015	Cancer Care Ontario, Canada	Fatigue, sexual dysfunction, quality of life, distress, depression, anxiety	Adults	Follow‐up care	Prostate cancer
14. Interventions to Address Sexual Problems in People with Cancer	2016	Cancer Care Ontario, Canada	Sexual dysfunction, body image, social support	Adults	Any stage of care	Any cancer type
15. Anxiety and Depression in Adult Cancer Patients	2023	European Society for Medical Oncology (ESMO), Switzerland	Anxiety, depression	Adults	Any stage of care	Any cancer type
16. Care of the Adult Cancer Patient at the End of Life	2021	European Society for Medical Oncology (ESMO), Switzerland	Existential/spiritual concerns, social support, fatigue, distress	Adults	Palliative care	Any cancer type
17. Cancer‐Related Fatigue: ESMO Clinical Practice Guidelines for Diagnosis and Treatment	2020	European Society for Medical Oncology (ESMO), Switzerland	Fatigue	No age specified	Any stage of care	Any cancer type
18. Psychosocial Care for Adult Cancer Patients: Guidelines of the Italian Medical Oncology Association	2021	Italian Medical Oncology Association, Italy	Distress, depression	Adults	Any stage of care	Any cancer type
19. Adolescent and Young Adult (AYA) Oncology	2018	National Comprehensive Cancer Network (NCCN), USA	Quality of life, social support, distress, depression, anxiety, existential/spiritual concerns	Young adults and adolescents	Any stage of care	Any cancer type
20. Cancer‐Related Fatigue	2015	National Comprehensive Cancer Network (NCCN), USA	Fatigue	No age specified	Any stage of care	Any cancer type
21. Distress Management	2019	National Comprehensive Cancer Network (NCCN), USA	Distress, depression, anxiety	No age specified	Any stage of care	Any cancer type
22. Palliative Care	2016	National Comprehensive Cancer Network (NCCN), USA	Existential/spiritual concerns, quality of life, distress, sleep disturbances	No age specified	Palliative care	Any cancer type
23. Survivorship	2017	National Comprehensive Cancer Network (NCCN), USA	Distress, depression, anxiety, fatigue, sexual dysfunction, sleep disturbances	No age specified	Follow‐up care	Any cancer type

The academic literature search yielded a total of 7498 relevant hits and identified 9 CPGs that met full eligibility criteria, only 1 of which were unique to this search (i.e., not identified by any other search method). In contrast, the gray literature search generated 2925 records and identified a total of 22 CPGs, 14 of which were unique to this search. Differences emerged between the three different types of gray literature searches. First, the targeted Internet search yielded 860 records and identified 21 CPGs in total, 1 of which was unique to this search. Next, the naïve Google search yielded 1098 records and identified 16 CPGs overall, 1 of which was unique to the search. Lastly, the Google Scholar search generated 900 records and identified 6 CPGs, all of which were also identified by another search. Refer to Table 2 for a visual representation of the identification of each CPG by the different search methods.

**TABLE 2:** Identification of psychosocial oncology clinical practice guidelines (CPGs) by each search method.

Clinical practice guideline (CPG)^a^	Academic literature search	Gray literature search: naïve Google search	Gray literature search: targeted internet search	Gray literature search: Google scholar search
Guideline 1	X		X	
Guideline 2	X		X	X
Guideline 3	X		X	
Guideline 4	X	X	X	
Guideline 5	X		X	X
Guideline 6			X	X
Guideline 7		X	X	
Guideline 8			X	
Guideline 9	X			
Guideline 10	[X]	X	X	
Guideline 11		X	X	X
Guideline 12	X	X	X	X
Guideline 13		X	X	
Guideline 14		X	X	
Guideline 15		X	X	
Guideline 16	X	X	X	X
Guideline 17	X	X	X	
Guideline 18		X		
Guideline 19	[X]	X	X	
Guideline 20	[X]	X	X	
Guideline 21	[X]	X	X	
Guideline 22	[X]	X	X	
Guideline 23	[X]	X	X	

*Note:* Brackets ([X]) indicate that only an outdated version of the CPG was found despite the availability of a newer version.

^a^ CPGs are numbered based on order in Table 1.

### 3.3 Characteristics of identified clinical practice guidelines

The 23 psychosocial oncology CPGs identified through the environmental scan were published and/or updated between 2014 and 2023 and were developed by multiple organizations, including the American Cancer Society, the American Society of Clinical Oncology (ASCO), the Canadian Association of Psychosocial Oncology (CAPO), Cancer Care Ontario, the European Society for Medical Oncology (ESMO), the National Comprehensive Cancer Network (NCCN), and the Italian Medical Oncology Association. The majority of the CPGs were developed in the USA (*n* = 14, 60.9%), followed by Canada (*n* = 5, 21.7%) and Europe (*n* = 4, 17.4%). The psychosocial symptoms or concerns addressed by the CPGs included distress (*n* = 15, 65.2%), depression (*n* = 15, 65.2%), fatigue (*n* = 11, 47.8%), anxiety (*n* = 10, 43.5%), social support (*n* = 8, 34.8%), sexual dysfunction (*n* = 7, 30.4%), existential/spiritual concerns (*n* = 6, 26.1%), quality of life (*n* = 5, 21.7%), sleep disturbances (*n* = 4, 17.4%), and body image preoccupations (*n* = 4, 17.4%). Approximately half of the CPGs targeted a single specific or limited range of symptoms (*n* = 11, 47.8%) whereas the other half addressed global psychosocial care, addressing a wider scope of potential symptoms and concerns (*n* = 12, 52.2%). See Table 1 for more detailed characteristics of the 23 relevant CPGs.

## 4 DISCUSSION

Clinical practice guidelines are essential tools for quality healthcare provision and clinical practice. They are meant to support a wide range of purposes, including informing professionals about new pharmaceutical and non‐pharmaceutical interventions, reducing the variability in clinical practice, improving patient‐reported outcomes, and establishing widely applicable clinical standards.^30^, ^31^, ^32^ The current study identified 23 up‐to‐date CPGs that provide recommendations for non‐pharmacological interventions in the treatment of the psychosocial symptoms of individuals diagnosed with cancer. Results revealed the academic literature search to be less efficient than the gray literature search at identifying CPGs, as evidenced by yielding fewer CPGs despite generating the highest number of relevant hits prior to screening. In contrast, the gray literature search yielded a greater number of CPGs missed by the academic search and identified all but one of the psychosocial oncology CPGs. In terms of the different gray literature search methods, the targeted Internet search was the most effective, identifying the greatest number of overall and unique CPGs. This suggests that clinicians looking for psychosocial oncology CPGs do not need to conduct a thorough, time‐consuming academic search; rather, they will be best served by conducting a targeted search through the websites of key organizations and special interest groups. The efficiency of this search method is especially important given its widespread accessibility and time effectiveness. However, our findings also highlight some key concerns regarding the current state of psychosocial oncology CPGs as evidence‐based tools.

The accessibility of CPGs through the gray literature search is encouraging when considering that clinicians report more using informal search methods to find information and tools more frequently than academic databases.^33^ However, an Internet search alone may not be sufficient. Our academic search identified several CPGs not detected by the gray literature search, meaning that even effective informal search methods may fail to identify relevant CPGs identifiable through a complementary database search. Yet, there are several barriers to conducting this type of search. Due to the high number of records generated, it would require a significant investment of time on the part of clinicians, who often face high work demands and lack time to conduct such a search. Further, even if clinicians were able and willing to conduct a database search, they are more likely to run the risk of identifying outdated versions of existing CPGs. Previously, clinicians could refer to the National Guidelines Clearinghouse (NGC)—a federally funded resource – which served as a repository for all CPGs. However, NGC is no longer operative due to loss funding in 2018.^34^ Since then, no new online repository for CPGs has been established. An online hub for all currently available CPGs, including psychosocial oncology CPGs, would play an essential role in addressing these barriers and ensuring ease of access for clinicians to a variety of CPGs ranging in focus (e.g., targeting a specific symptom or providing more global care recommendations), organization of origin, country of development, and methods of assessing evidence.

The limited availability of up‐to‐date CPGs through peer‐reviewed sources also raises concerns about their quality. The peer‐review component of journal publication allows for additional independent appraisal and oversight into CPG development, providing an opportunity for improvements to the guideline content and recommendations prior to publication.^35^ Without an independent, external assessment of the quality and methodological rigor, it is unclear whether encouraging the use of these CPGs represents a step towards evidence‐based practice and optimal patient care. As such, even if many of these CPGs are easily accessible, clinicians may face a dilemma where they wish to be more evidence‐based in their practice but are unsure about the quality and/or applicability of the CPGs they find. Thus, implementation and adherence of CPGs may be well‐served by publication in a peer‐reviewed journal, given their function as a form of “quality control” for many institutions, clinicians, or other stakeholders. Yet, guideline developers may neglect to publish newly developed or updated CPGs in peer‐reviewed journals for a variety of reasons. For example, the formulation of recommendations by the guideline development team emerged from an extensive review process by content experts; peer‐reviewers and editors may be unaware of the full range of considerations and, consequently, suggested changes and edits to recommendations may fail to account for the full scope of relevant information. In addition, engaging in the peer‐review process is time‐consuming and significant time delays between each stage of submission and revision may result in the findings and recommendations of the CPG already being outdated by the time it is published.^35^

The risk of outdated guidelines is a more overarching concern. The most common reason for exclusion in our study was due to guidelines failing to be updated or revised within the past 10 years, meaning that recommendations will often fail to translate the latest empirical findings.^35^ This is coherent with previous findings that guidelines often fail to be updated, despite overarching recommendations to review CPGs for updates every 3 years.^35^, ^36^, ^37^ This limits their applicability as guides for bridging the science‐practice gap. Even a high quality and helpful CPG is no longer well‐suited for use as an evidence‐based tool if its findings are outdated, despite the large amounts of financial and non‐financial resources invested into its conception and development.^38^ The lack of updating also increases the risk that resources will be invested into the creation of new CPGs by different organizations, thus duplicating the previous work of guidelines development teams. While some government‐funded organizations have enough resources to adhere to more rigorous and structured updating procedures (e.g., the National Comprehensive Cancer Network (NCCN)), it is important to acknowledge many institutions face important barriers to the regular appraisal and updating of existing CPGs.^38^

### 4.1 Limitations

This CPG environmental scan includes several limitations. All identified CPGs were developed by Western organizations in high‐income countries (North America and Europe). Although there were no geographical constraints on the search, this might be a consequence of restricting search results to English language results only. Consequently, the recommendations of the CPGs identified most likely reflect the cultural norms of these countries and are designed for application within their healthcare systems and cultural contexts, which could limit their usefulness for clinicians practicing in different countries. Although various organizations have spearheaded efforts to produce CPG adaptations to address the specific needs of their regions,^39^, ^40^ the identification of such adaptation was beyond the scope of this study. There may also be additional psychosocial oncology CPGs not accessible through the methodology used herein. Clinicians working in psychosocial oncology settings may have exclusive access to CPGs through their own professional networks and resources (e.g., CPGs developed for exclusive use by employees of a particular hospital network). As such, the current findings may not reflect the psychosocial oncology CPGs most directly accessed or used by clinicians.

### 4.2 Future directions and conclusions

Our findings suggest that psychosocial oncology CPGs address a wide range of symptoms and concerns, are accessible to clinicians, and can be found through even informal search methods. Several concerns about the utility of these CPGs as evidence‐based tools arose. Most notably, many existing psychosocial oncology CPGs fail to be updated following their initial publication and little information is available about their quality. Future research should seek to evaluate the methodological quality of currently available psychosocial oncology CPGs. In addition, our findings do not inform us on the current state of CPG use by clinicians providing psychosocial care to individuals diagnosed with cancer. Previous research found that clinicians often do not integrate evidence‐based interventions into their service delivery and report limited knowledge about CPGs as a whole.^38^, ^41^ A more in‐depth understanding of the current clinical use and implementation of psychosocial oncology CPGs would more effectively address barriers to their use and further support efforts to mobilize resources to keep CPGs up‐to‐date.

## AUTHOR CONTRIBUTIONS

**Catherine Bergeron:** Conceptualization (lead); data curation (lead); formal analysis (lead); methodology (lead); project administration (lead); writing – original draft (lead); writing – review and editing (lead). **Michelle Azzi:** Data curation (supporting); formal analysis (supporting); project administration (supporting); writing – review and editing (supporting). **Adina Coroiu:** Formal analysis (supporting); methodology (supporting); writing – original draft (supporting); writing – review and editing (supporting). **Carmen G. Loiselle:** Investigation (supporting); methodology (supporting); supervision (supporting); writing – review and editing (supporting). **Martin Drapeau:** Investigation (supporting); supervision (supporting); writing – review and editing (supporting). **Annett Körner:** Conceptualization (equal); formal analysis (supporting); funding acquisition (lead); investigation (supporting); methodology (supporting); resources (supporting); supervision (supporting); writing – original draft (supporting); writing – review and editing (supporting).

## CONFLICT OF INTEREST STATEMENT

The authors declare they have no conflict of interests.

## Data Availability

The data for the present study is available in the content of the article and Appendix, Table A1. Further inquiries may be directed to the corresponding author.

## Appendix

**TABLE A1:** Keywords and search strategy by database.

Database	Keywords and search strategy
Medline (1946‐)	(((psycho*.mp OR supportive care.mp OR mental health.mp OR well‐being.mp) AND (oncology.mp OR cancer.mp)) OR *Psycho‐Oncology/ [MeSH]) AND (*Practice Guideline/ [MeSH] OR *Guideline/ [MeSH] OR guideline*.mp)
PubMed	(((((((psycho*[Text Word]) OR (supportive care[Text Word])) OR (mental health[Text Word])) OR (well‐being[Text Word])) AND (oncolog*[Text Word])) OR (cancer[Text Word])) OR (psycho‐oncolog*[Text Word])) AND (guideline*[Publication Type])
PsycINFO (1987‐)	(((*Psychosocial Factors/ [MeSH] OR psycho*.mp OR supportive care.mp OR mental health.mp OR *Mental Health/ [MeSH] OR well‐being.mp) AND (oncolog*.mp OR *Oncology/ [MeSH] OR cancer.mp)) OR psycho‐oncology.mp) AND (*Treatment Guidelines/ [MeSH] OR guideline*.mp OR practice guideline*.mp)
JSTOR	(psycho‐ OR supportive care OR mental health OR well‐being) AND (oncology‐ OR cancer) AND (practice guideline‐ OR guideline‐)
Clinical Key	(psychosocial oncology AND guideline [Publication Type]) OR (Psycho‐oncology AND guideline [Publication Type]) OR (supportive care cancer AND guideline [Publication Type])

*Note*: Truncation to find alternative spellings variations is indicated by an asterisk or hyphen after a keyword. An asterisk before a keyword designates the theme as a major topic of the article.
